# Police and public health partnerships: Evidence from the evaluation of Vancouver's supervised injection facility

**DOI:** 10.1186/1747-597X-3-11

**Published:** 2008-05-07

**Authors:** Kora DeBeck, Evan Wood, Ruth Zhang, Mark Tyndall, Julio Montaner, Thomas Kerr

**Affiliations:** 1 British Columbia Centre for Excellence in HIV/AIDS, 608-1081 Burrard Street, Vancouver, B.C., V6Z 1Y6, Canada; 2 Department of Medicine, University of British Columbia, Vancouver, Canada

## Abstract

In various settings, drug market policing strategies have been found to have unintended negative effects on health service use among injection drug users (IDU). This has prompted calls for more effective coordination of policing and public health efforts. In Vancouver, Canada, a supervised injection facility (SIF) was established in 2003. We sought to determine if local police impacted utilization of the SIF. We used generalized estimating equations (GEE) to prospectively identify the prevalence and correlates of being referred by local police to Vancouver's SIF among IDU participating in the Scientific Evaluation of Supervised Injecting (SEOSI) cohort during the period of December 2003 to November 2005. Among 1090 SIF clients enrolled in SEOSI, 182 (16.7%) individuals reported having ever been referred to the SIF by local police. At baseline, 22 (2.0%) participants reported that they first learned of the SIF via police. In multivariate analyses, factors positively associated with being referred to the SIF by local police when injecting in public include: sex work (Adjusted Odds Ratio [AOR] = 1.80, 95%CI 1.28 – 2.53); daily cocaine injection (AOR = 1.54, 95%CI 1.14 – 2.08); and unsafe syringe disposal (AOR = 1.46, 95%CI 1.00 – 2.11). These findings indicate that local police are facilitating use of the SIF by IDU at high risk for various adverse health outcomes. We further found that police may be helping to address public order concerns by referring IDU who are more likely to discard used syringes in public spaces. Our study suggests that the SIF provides an opportunity to coordinate policing and public health efforts and thereby resolve some of the existing tensions between public order and health initiatives.

## Background

In various urban settings, street-level policing practices targeting drug related public disorder, such as open drug dealing and drug consumption, have been shown to interrupt health service use by injection drug users (IDU) [1,2]. Specifically, pressures introduced by street level police crackdowns have been found to displace IDU away from needle exchange programs and other specialized HIV prevention and health promotion services, as well as exacerbate risky injection practices among street injectors including rushing injections and injecting with used syringes [3-7]. This has prompted calls for more effective coordination of policing and public health initiatives [8-10].

In Vancouver, Canada, local street level policing practices have similarly been found to complicate HIV prevention initiatives in some instances [11-13]. However, the local Vancouver Police Department supported the opening of a pilot supervised injection facility (SIF) in Vancouver in September 2003 and subsequently adopted the strategy of actively encouraging individuals found injecting in public to attend the local SIF [14]. Past evaluations of SIFs in other settings indicate that police support plays an important role in the successful operation of these facilities [15], however, we know of no studies which have specifically examined police referrals and their impact on facilitating access to SIFs. Given the continued call for more effective policing-public health partnerships [16,17] we sought to determine if local police were facilitating the use of Vancouver's SIF.

## Methods

The current analysis is based on longitudinal data derived from the Scientific Evaluation of Supervised Injecting (SEOSI) cohort which is a representative sample of supervised injection facility users. This study has been described in detail previously [18,19]. Briefly, beginning December 2003, randomly selected SIF clients were recruited into SEOSI. At baseline and semi-annually participants provide blood samples and complete an interviewer-administered questionnaire. The questionnaire elicits demographic data as well as information about drug use patterns, HIV risk behavior, access to health and social services, SIF use, and interactions with local police and criminal justice systems. All participants provide written informed consent and are given a $20 honorarium at each study visit. The study has received ethical approval from St. Paul's Hospital and the University of British Columbia's Research Ethics Board.

To explore the role of local police in supporting use of Vancouver's SIF we assessed the proportion of participants who reported first learning of the SIF via communication with police. In addition, we asked participants at baseline and at each study follow-up if local police had helped them find the SIF, or taken them there when they were injecting in public. To identify the population most affected by this policing strategy we conducted longitudinal analysis of factors associated with reporting having been referred to the SIF by local police. For this we included all participants seen for baseline and follow-up interviews during the period of December 2003 to December 2005. Given that policing practices are known to exacerbate high-risk injecting among IDU who inject in public spaces [3-6,11], the dependent variable for the present study was based on self-report and was defined only as having been referred to the supervised injection facility by police when injecting in public in the last six months. Other variables of interest included socio-demographic information: age (per year older), gender (female vs. male), Aboriginal ethnicity (yes vs. no) and homelessness, defined as having no fixed address for the last six months (yes vs. no). Drug use variables considered refer to behaviours in the past six months and included: frequent heroin injection (≥ daily vs. < daily), frequent cocaine injection (≥ daily vs. < daily), borrowing and lending used syringes (yes vs. no), and unsafe syringe disposal, defined as having dropped a syringe outdoors after using it (yes vs. no). Another characteristic considered was involvement in sex work in the last six months (yes vs. no).

Since analyses of factors potentially associated with having been referred to the SIF by police included serial measures for each participant, we used generalized estimating equations (GEE) for binary outcomes with logit link for the analysis of correlated data to determine factors associated with referrals to the SIF throughout the 24-month follow-up period. These methods provided standard errors adjusted by multiple observations per person using an exchangeable correlation structure. Therefore, data from every participant follow-up visit was considered in this analysis. This approach has been used successfully in previous analysis [20,21]. As a first step, we used univariate GEE analyses to determine factors associated with having been referred to the injection facility by police. All variables that were *p *< 0.05 in GEE univariate analyses were then entered in a multivariate logistic GEE model. All statistical analyses were performed using SAS software version 9.1 (SAS, Cary, NC). All p-values are two sided.

## Results

A total of 1090 participants were recruited during the study period, including 317 (29.1%) women and 211 (19.4%) persons of Aboriginal ancestry. The median age of participants was 38.4 years (IQR = 32.7–44.3) at baseline. This sample contributed 3083 observations and the median number of study visits was 3 (IQR = 2–4). A total of 182 (16.7%) participants reported having been referred to the SIF by police at some point during the study period. At baseline, 22 (2.0%) participants reported that they first learned of the SIF via communication with local police.

The univariate GEE analyses of factors associated with having been referred to the SIF by local police are presented in Table 1. Factors found to be associated with having been referred to the SIF by local police in univariate analyses included: older age (odds ratio [OR] = 0.98, 95% confidence interval [CI] 0.96–1.00); Aboriginal ethnicity (OR = 1.51, 95%CI 1.05–2.16); homelessness (OR = 1.49, 95%CI 1.08–2.06); sex work (OR = 2.03, 95%CI 1.46–2.83); frequent heroin injection (OR = 1.53, 95%CI 1.14–2.06); frequent cocaine injection (OR = 1.66, 95%CI 1.24–2.24); and unsafe syringe disposal (OR = 1.73, 95%CI 1.20 – 2.50).

**Table 1:** Univariate and multivariate GEE^a ^analyses of factors associated with being referred to Vancouver's supervised injection facility by local police officers

**Characteristic^f^**	**OR^b ^(95% CI^d^)**	***p-value*^c^**	**AOR^b ^(95% CI^d^)**	***p-value***
**Older Age**				
per year older	0.98 (0.96 – 1.00)	0.041	1.00 (0.98 – 1.02)	0.961
**Gender**				
Female vs. Male	0.73 (0.52 – 1.01)	0.059		
**Aboriginal Ethnicity**				
Yes vs. No	1.51 (1.05 – 2.16)	0.027	1.41 (0.99 – 2.03)	0.065
**Homelessness ^e^**				
Yes vs. No	1.49 (1.08 – 2.06)	0.014	1.28 (0.92 – 1.78)	0.140
**Sex Work ^e^**				
Yes vs. No	2.03 (1.46 – 2.83)	<0.001	1.80 (1.28 – 2.53)	<0.001
**Frequent Heroin Injection ^e^**				
≥ daily vs. < daily	1.53 (1.14 – 2.06)	0.005	1.32 (0.98 – 1.79)	0.070
**Frequent Cocaine Injection ^e^**				
≥ daily vs. < daily	1.66 (1.24 – 2.24)	<0.001	1.54 (1.14 – 2.08)	0.005
**Syringe Sharing ^e^**				
Yes vs. No	0.99 (0.68 – 1.44)	0.971		
**Unsafe Syringe Disposal ^e^**				
Yes vs. No	1.73 (1.20 – 2.50)	0.004	1.46 (1.00 – 2.11)	0.048
				

Note: ^a^GEE = Generalized Estimating Equation; ^b^OR = Odds Ratio, AOR = Adjusted Odds Ratio; ^c^Values based on Wald χ^2 ^with 1 degree of freedom; ^d^CI = Confidence Interval; ^e^Denotes activities or situations referring to the previous 6 months; ^f^For full variable definitions see methods section.

In the multivariate GEE analysis, also shown in Table 1, factors that remained independently associated with having been referred to the SIF by local police included: sex work (adjusted odds ratio [AOR] = 1.80, 95%CI 1.28 – 2.53); frequent cocaine injection (AOR = 1.54, 95%CI 1.14 – 2.08); and unsafe syringe disposal (AOR = 1.46, 95%CI 1.00 – 2.11).

## Discussion

In the present study, we found that approximately 17% of participants reported having been referred to the SIF by Vancouver police officers when injecting in public and those engaged in sex work and frequent cocaine injection were more likely to be referred. Given the criminalization of sex work in Canada, the association between sex work and police referrals may be a reflection of sex worker's higher exposure to police. Other research in this setting has documented that interactions between sex workers and police are frequent and at times violent. In addition, contact with police was found to displace sex workers to isolated industrial areas where their ability to protect themselves from violence and HIV risk was severity compromised [22]. However, by referring IDU engaged in sex work and frequent cocaine injection to a health focused facility, local police are likely helping to reduce health-related harms by reaching IDU at heightened risk for adverse health outcomes, including HIV infection and violence [22,23]. Further, by referring IDU who engage in unsafe public syringe disposal to the SIF, police may also be helping to reduce the public order impacts of public injecting.

Collectively, these contributions suggest that the Vancouver SIF is providing local police with a mechanism to address public injection drug use in a manner that promotes public safety and appears to resolve some of the existing tensions between public health and public order initiatives. Given previously documented tensions between police and other public health initiatives in this setting [11-13], the ability of SIFs to promote public order objectives may help to explain why local police have been supportive of this particular program. In fact, research conducted for the Canadian Expert Advisory Committee on Supervised Injection Site Research found that the majority of local Vancouver police officers interviewed support the Vancouver SIF as means of improving public order [24]. Despite clear support for the Vancouver SIF by local police officers, external national law enforcement bodies remain vocally opposed to the facility. Most recently the Canadian Police Association (CPA) issued a public call for the Government of Canada to "shut down the failed Supervised Injection Site experiment" and suggested that most police officers do not support the initiative [25,26]. These statements highlight a disconnect between the views of police officers working in direct proximity to the SIF and those of external law enforcement organizations.

In other settings with SIFs, police support appears to be similarly connected with public order objectives and police typically partner with local services providers, residents and business to ensure the successful operation of SIFs [15]. Past evaluations of European SIFs highlight the importance of obtaining police support for these initiatives as policing practise in areas surrounding SIFs have been found to have considerable impact on the operation of, and public support for, these facilities. For example, police crackdowns on open drug scene and the potential for drug market activity to re-emerge in the vicinity of a SIF were identified as forces that have undermined public support for SIFs [15]. The importance of coordinating efforts among police, service providers and other stakeholder is widely acknowledged, however, documentation of successful policing approaches around SIFs, such the current example of police referring IDU injecting in public to the Vancouver SIF, warrants further exploration.

While the findings of the present study suggest that local police are promoting use of the Vancouver SIF it should be noted that in a prior study it was found that 5% of local IDU reported having been deterred from using the SIF due to police presence around the facility [27]. Still, while local police presence may limit access to the SIF for some, overall findings indicate that they are helping to facilitate access. Regardless, in order to promote optimal access to the SIF, additional efforts, including further research, should be undertaken to determine how particular services barriers can be addressed.

Despite these positive findings, the extent to which police are able to address public drug use by directing injectors to the local SIF is largely constrained by the limited seating capacity and operating hours of the 12 seat pilot facility [27]. In addition, the SIF does not accommodate crack cocaine smoking which is a central contributing factor to current drug-related street disorder [28]. While the SIFs has been shown to effectively reduce rates of syringe sharing, increase entry to detoxification services and improve public order in the area [29-31], it is clear that one small intervention cannot meaningfully address public drug use in Vancouver and its potential to eradicate the public drug scene should not be overstated.

There are several potential limitations in the study to be noted. Primarily, this study relied on self-reported information concerning stigmatized behaviours, such as public drug use and syringe disposal and hence is susceptible to socially desirable reporting [32]. In the present study this may have led to an under-reporting of unsafe syringe disposal and other stigmatized behaviours. In addition, policing presence may encourage use of the SIF among people not directly referred and this study does not account for this positive effect on public order. In turn, our findings are likely conservative and may perhaps under-represent the impact that local police are having on use of the facility.

Our findings indicate that local police are facilitating use of the SIF by IDU at heightened risk for various adverse health outcomes. These data further suggest that police may be helping to address public order concerns by referring IDU who are likely to discard used syringes in public spaces. Therefore, the SIF appears to provide an opportunity to coordinate policing and public health efforts and thereby resolve some of the existing tensions between public order and health initiatives.

## Competing interests

The authors declare that they have no competing interests.

## Authors' contributions

The specific contributions of each author are as follows: KD and TK were responsible for study design and prepared the first draft of the analysis; RZ conducted the statistical analyses; EW, MT and JM contributed to the main content and provided critical comments on the final draft. All authors approved the final manuscript.

## Funding

The evaluation of the supervised injecting facility was originally made possible through a financial contribution from Health Canada, although the views expressed herein do not represent the official policies of Health Canada. The evaluation is currently supported by the Canadian Institutes of Health Research and Vancouver Coastal Health. TK is supported by the Michael Smith Foundation for Health Research and the Canadian Institutes of Health Research. KD is supported by a Canadian Institutes of Health Research Doctoral Research Award and a Michael Smith Foundation for Health Research Senior Graduate Trainee Award. Funding agencies had no role in study design, data collection, analysis or writing of the report, nor did they have a role in the decision to submit the paper for publication.

## References

[B1] Kerr T, Small W, Wood E (2005). The public health and social impacts of drug market enforcement: A review of the evidence. International Journal of Drug Policy.

[B2] Cohen J, Csete J (2006). As strong as the weakest pillar: Harm reduction, law enforcement and human rights. International Journal of Drug Policy.

[B3] Cooper H, Moore L, Gruskin S, Krieger N (2005). The impact of a police drug crackdown on drug injectors' ability to practice harm reduction: A qualitative study. Social Science & Medicine.

[B4] Aitken C, Moore D, Higgs P, Kelsall J, Kerger M (2002). The impact of a police crackdown on a street drug scene: Evidence from the street. International Journal of Drug Policy.

[B5] Maher L, Dixon D (1999). Policing and public health: Law enforcement and harm minimization in a street-level drug market. British Journal of Criminology.

[B6] Burris S, Blankenship KM, Donoghoe M (2004). Addressing the "risk environment" for injection drug users: The mysterious case of the missing cop. Milbank Q.

[B7] Blankenship K, Koester S (2002). Criminal law, policing policy, and HIV risk in female street sex workers and injection drug users. J Law Med Ethics.

[B8] Burris S, Strathdree S (2006). To serve and protect? Towards a better relationship between drug control policy and public health. AIDS.

[B9] Blankenship K, Smoyer A, Ed (2004). Public health, research, and law enforcement: The case of HIV/AIDS prevention.

[B10] Caulkins J (2002). Law enforcement's role in a harm reduction regime. Crime and Justice Bulletin 64.

[B11] Small W, Kerr T, Charette J, Schechter MT, Spittal PM (2006). Impacts of intensified police activity on injection drug users: Evidence from an ethnographic investigation. International Journal of Drug Policy.

[B12] Wood E, Kerr T, Small W, Jones J, Schechter MT, Tyndall MW (2003). The impact of a police presence on access to needle exchange programs. J Acquir Immune Defic Syndr.

[B13] Wood E, Spittal P, Small W, Kerr T, Li K, Hogg R, Tyndall M, Montaner J, Schechter M (2004). Displacement of Canada's largest public illicit drug market in response to a police crackdown. Canadian Medical Association Journal.

[B14] Vancouver Police Department (2006). Vancouver Police Department drug policy.

[B15] Hedrich D (2004). European report on drug consumption rooms.

[B16] Mazerolle L, Soole DW, Rombouts S (2006). Street-level drug law enforcement: A meta-analytical review. Journal of Experimental Criminology.

[B17] Mazerolle L, Soole D, Rombouts S (2007). Drug law enforcement: A review of the evaluation literature. Police Quarterly.

[B18] Wood E, Kerr T, Lloyd-Smith E, Buchner C, Marsh DC, Montaner JSG, Tyndall MW (2004). Methodology for evaluating Insite: Canada's first medically supervised safer injection facility for injection drug users. Harm Reduction Journal.

[B19] Wood E, Kerr T, Montaner JS, Strathdee SA, Wodak A, Hankins CA, Schechter MT, Tyndall MW (2004). Rationale for evaluating North America's first medically supervised safer-injecting facility. Lancet Infect Dis.

[B20] Kerr T, Marsh D, Li K, Montaner J, Wood E (2005). Factors associated with methadone maintenance therapy use among a cohort of polysubstance using injection drug users in Vancouver. Drug Alcohol Depend.

[B21] Shah N, Celentano D, Vlahov D, Stambolis V, Johnson L, Nelson K, Strathdee S (2000). Correlates of enrollment in methadone maintenance treatment programs differ by HIV-serostatus. AIDS.

[B22] Shannon K, Kerr T, Allinott S, Chettiar J, Shoveller J, Tyndall MW (2008). Social and structural violence and power relations in mitigating HIV risk of drug-using women in survival sex work. Social Science & Medicine.

[B23] Tyndall M, Spittal P, Laliberte N, Li K, O'Shaughnessy M, Schechter M (2002). Intensive injection cocaine use as a primary risk factor of HIV seroconversion among polydrug users in Vancouver. AIDS.

[B24] Canadian Police Association CPA urges government to shut down failed supervised injection site experiment [press release].

[B25] Ogborne A, Larke B, Plecas D, Waller I, Rehm J (2008). Vancouver's INSITE service and other supervised injection sites: What has been learned from research? Final report of the expert advisory committee on supervised injection site research.

[B26] Canadian Police Association (2007). 2007 CPA Political Agenda. Express.

[B27] Petrar S, Kerr T, Tyndall MW, Zhang R, Montaner JS, Wood E (2007). Injection drug users' perceptions regarding use of a medically supervised safer injecting facility. Addictive Behaviors.

[B28] Shannon K, Ishida T, Morgan R, Bear A, Oleson M, Kerr T, Tyndall M (2006). Potential community and public health impacts of medically supervised safer smoking facilities for crack cocaine users. Harm Reduction Journal.

[B29] Kerr T, Tyndall M, Li K, Montaner J, Wood E (2005). Safer injection facility use and syringe sharing in injection drug users. The Lancet.

[B30] Wood E, Tyndall M, Montaner J, Kerr T (2006). Summary of findings from the evaluation of a pilot medically supervised safer injecting facility. Canadian Medical Association Journal.

[B31] Wood E, Tyndall M, Zhang R, Stoltz J, Lai C, Montaner J, Kerr T (2006). Attendance at supervised injecting facilities and use of detoxification services. New England Journal of Medicine.

[B32] Des Jarlais D, Paone D, Milliken J, Turner C, Miller H, Gribble J, Shi Q, Hagan H, Friedman S (1999). Audio-computer interviewing to measure risk behaviour for HIV among injecting drug users: A quasi-randomised trial. Lancet.

